# Current situation and countermeasures of traditional Chinese medicine resource distribution: a case study of Wuyi County in China

**DOI:** 10.3389/fpubh.2025.1473833

**Published:** 2025-04-17

**Authors:** Xiao Zhu, Zhiqiang Zhang, Lingjian Xu, Xiayuan Zhong

**Affiliations:** Zhejiang Province Chinese Medicine Hospital of Wuyi County, Wuyi, China

**Keywords:** traditional Chinese medicine resource, distribution status, Wuyi mode, countermeasure, development prospect

## Abstract

**Objective:**

To investigate the current status of Traditional Chinese Medicine (TCM) resource development within Chinese counties and identify enforceable strategies.

**Methods:**

Wuyi County was adopted as a case study, analyzing the distribution of TCM resources within the county. Based on the TCM development models employed in other counties, this study proposed the “Wuyi mode,” which integrates “medical consortiums + medical communities + specialty alliances + shared Chinese medicine pharmacies.” Meanwhile, the model was applied in practice, and its effectiveness was verified.

**Results:**

The study provides novel insights into addressing the issue of uneven distribution of TCM resources at the county level and contributes to the establishment of a new model for TCM development.

**Conclusion:**

The “Wuyi mode” offers a valuable framework that may inform the development of TCM in other counties across China and potentially serve as a reference for TCM development globally.

## 1 Introduction

Traditional Chinese Medicine (TCM) is a valuable cultural heritage of China, making substantial contributions throughout history and in contemporary times to the health, well-being, and prosperity of the Chinese nation. It embodies the accumulated wisdom of the Chinese people in their long-standing struggle against diseases. According to available data (1), TCM has gained recognition in 29 countries and regions, including Australia, the Netherlands, and Singapore. Eighteen countries and regions have integrated TCM into their healthcare insurance systems. Chinese herbal medicine is registered in countries such as the United Kingdom, Sweden, and Russia. With the increasing recognition of the therapeutic efficacy of TCM, international research on TCM is expanding. During the 13th Five-Year Plan period, the strategic framework for TCM development was vigorously advanced and refined. The policy environment was consistently optimized, significantly enhancing the support for TCM.

TCM, revered as a treasure of the Chinese nation with a long history, embodies profound knowledge. However, despite multiple opportunities, such as national policy support and burgeoning public demand for TCM services, the distribution and utilization of TCM resources encounter numerous challenges (2). In March 2022, the State Council issued the “14th Five-Year Plan for the Development of Traditional Chinese Medicine,” pinpointing persistent imbalances and inadequacies in TCM advancement (3). It reveals the shortage of high-quality medical service resources in TCM and the relatively weak TCM service infrastructure at the grassroots level (4). Disparities in TCM development across regions are pronounced, manifested in some areas boasting ample TCM service resources while others languish in scarcity. Relevant studies have shown a geographical gradient in health resource aggregation across China, decreasing from east to west. The eastern region exhibits the highest concentration, followed by the central region, and the western region trails with the lowest (5). This imbalance engenders inefficiencies in resource allocation and affects the popularization and equity of TCM services, depriving residents in some areas of access to high-quality TCM services.

This article takes Wuyi County as a case study. With the support of the health administrative department, a comprehensive investigation into the distribution of TCM resources within the county was conducted. Drawing on established models of TCM development from other counties, the “Wuyi mode” was devised and implemented, integrating a framework of “medical consortiums + medical communities + specialty alliances + shared Chinese medicine pharmacies.” This model yielded notable successes, offering valuable reference for the development of TCM in other counties, as well as in international contexts. This model provides significant practical implications.

## 2 Available knowledge

### 2.1 Current situation outside China

According to a review of international literature, although research specifically on grassroots TCM services is rare, mature integrated medical service systems exist. The World Health Organization defines integrated healthcare as the management and organization of integrated services, providing residents with easily accessible and acceptable health services, curbing health service expenditures, and ultimately achieving desired service outcomes (6). González-Ortiz et al. (7) believed that the primary purpose of integrated healthcare was to mitigate the fragmentation of health services, promote comprehensive medical care, and align medical care with individual needs, reflecting the importance of patient-centered care.

The United States and the United Kingdom exemplify pioneering models of integrated medical and health services. The United States has over a hundred medical groups. For instance, the Kaiser Permanente medical group in the United States caters to 9.6 million members. It was initially established to address emergency medical needs and provide continuous medical services to patients (8). Moreover, through the Health Insurance Marketplace of the Patient Protection and Affordable Care Act (ACA), the United States has improved healthcare experiences, reduced cost barriers, and enhanced the public’s participation in the healthcare system (9). Integrated Care Organization (ICO), like the Boston Community Medical Group in the United States, strives to streamline care delivery for patients prone to medical interruptions or reliant on emergency departments. The group seeks to integrate the fragmented medical care these patients often receive, thus saving costs. As a corresponding hospital network, ICO can provide personalized medical services to these patients (10). Furthermore, the United States has fostered a robust network of family physicians.

As for the United Kingdom, the National Health Service (NHS) provides free medical care to all citizens. However, it has become overwhelmed due to financial and population pressures. Subsequently, Clinical Commissioning Groups (CCGs) were established in 2012 to commission most NHS services of hospitals and communities in the local area. Integrated care systems (ICSs), also known as medical consortia, were built to alleviate the immense pressure derived from NHS funding constraints and population growth and aging. These systems aim to enhance community and social healthcare outside hospitals while coordinating general practice, community services, and hospital facilities to meet patient needs (11).

Japan’s healthcare system falls somewhere between those of the United Kingdom and the United States. While implementing universal healthcare coverage, Japan allows for the coexistence of privatized and personalized medical services. Its healthcare system comprises three types: national, public, and private. Similar to China, Japan has also established a three-tier healthcare system based on geographical location and socio-economic conditions. The first level (primary healthcare institutions) provides routine diagnosis, treatment, and rehabilitation for common and chronic diseases. The second (secondary healthcare institutions) offers specialized treatment for complex diseases. The third addresses rare and intricate conditions and supports some research and education (12).

Germany’s medical consortium service adopts a more targeted model of healthcare resource integration, establishing Disease Management Programs (DMP) specifically for chronic disease patients. These patients are entitled to receive personalized, comprehensive care with a focus on prevention and quality of life. The objective is to improve population health, patient orientation, and care quality while reducing per capita costs. The German healthcare system is primarily structured into four types: the family doctor system for initial consultations (Type 1); hospitals for inpatient care (Type 2); rehabilitation centers for post-discharge recovery (Type 3); and nursing institutions for older adult and mobility-impaired patients (Type 4). A separation of diagnosis and hospitalization system is employed. Hospitals provide inpatient services, separating treatment from medicine. Neither hospitals nor clinics have pharmacies, and only pharmacies are authorized to sell medications. In order to effectively manage patient referrals, the government encourages patients to first consult with family doctors for initial diagnoses. If further diagnosis or surgical treatment is necessary, patients are referred to specialized clinics or hospitals. Upon completion of hospital treatment, patients are directed to affiliated rehabilitation centers for continued recovery until full rehabilitation is achieved (13).

### 2.2 Current situation in China

The current landscape of China’s medical service system integration can be classified into four types:

1. Urban medical consortia: Spearheaded by a tertiary hospital, this mode unites multiple secondary hospitals, rehabilitation hospitals, nursing homes, and community health service centers to form “1 + X” medical consortia.
2. County-level medical consortia: Based on the county-township integrated management with “county hospitals as the leader, township health centers as the hub, and village clinics as the foundation,” this mode establishes county-level medical service systems with three-tier linkage among counties, townships, and villages.
3. Specialized alliances: Centered around specialized departments of one medical institution, this mode collaborates with other medical institutions possessing similar specialized technical capabilities to establish several specialized centers within a region.
4. Remote collaboration medical networks: Established by leading units in partnership with medical institutions in grassroots, remote, and underdeveloped areas, this mode vigorously promotes the construction of telemedicine service systems tailored to these areas. It propels secondary and tertiary hospitals to extend remote medical services to grassroots medical and health institutions.

The establishment of the Nanjing Gulou Hospital Group in 1996 marked the inception of China’s inaugural medical consortium (6). In August 2015, Shenzhen Luohu initiated the construction of medical consortia. The Luohu Hospital Group was founded to implement patient-centered integrated medical care. It unified group management, strengthened cooperation between community health stations and district-level hospitals, and achieved a mutually beneficial scenario for hospitals, doctors, and patients through government compensation, performance management, price adjustment, and drug procurement measures. The Luohu mode has promoted the transformation from a hospital-centered and treatment-focused medical system to a people-centered and community-based integrated medical system (14).

Gao et al. (15) examined the allocation of TCM resources and regional disparities across 31 provinces and municipalities in China from 2013 to 2019, identifying the primary factors influencing resource distribution. The findings indicated that the equity of TCM resource allocation was notably poorer in the eastern provinces, with marked disparities across regions. The eastern provinces have rigorously implemented policies such as the “Several Opinions of the State Council on Supporting and Promoting the Development of Traditional Chinese Medicine,” leading to swift advancements in TCM in these regions. These efforts have facilitated a more balanced integration of Chinese and Western medicine in eastern regions. Inner Mongolia’s long-standing history of growing medicinal herbs and its rich Mongolian medical heritage have laid a robust foundation for TCM development. Combined with strong local governmental support, Inner Mongolia ranks among the leading regions in terms of per capita TCM resource allocation. Overall, the level of TCM resource allocation in China is on the rise, with the eastern regions experiencing the fastest growth. However, negative growth has been observed in the eastern, central, and northeastern regions. The disparities in resource allocation can primarily be attributed to internal variations within the four major economic regions. Therefore, it is essential to delve into the underlying causes of regional discrepancies in TCM resource distribution.

In November 2022, the National Administration of Traditional Chinese Medicine (NATCM) issued the “14th Five-Year Plan for the Development of TCM Informatization,” which proposed that medical consortiums take a leading role, with TCM hospitals serving as key drivers of technological advancements. The plan emphasizes the exploration of telemedicine centers and shared Chinese medicine pharmacies, providing standardized remote medical services and TCM care. Additionally, the integration of mobile internet and big data technologies is intended to improve hierarchical medical systems, fostering medical information sharing and healthcare service collaboration. In alignment with this vision, several provinces have introduced models such as medical consortiums, shared Chinese medicine pharmacies, and specialty alliances at the county level. By the end of 2023, in D City, a major urban area in western China, nearly 200 medical consortium units were contracted by tertiary hospitals. A series of initiatives have been implemented to facilitate the downward flow of high-quality medical resources: first, efforts to attract prestigious medical institutions such as Huaxi Dazhou Women’s and Children’s Hospital, Sichuan Provincial People’s Hospital, and Dazhou First People’s Hospital; second, the introduction of doctoral experts from Beijing Jishuitan Hospital, Huaxi Hospital, Beijing Zhong-Ri Friendship Hospital, and the affiliated hospitals of Chongqing Medical University to D City, accelerating the aggregation of medical talent; third, the promotion of “Internet+” technologies, establishing a remote consultation system centered around Sichuan University Huaxi Hospital, Sichuan Provincial People’s Hospital, and designated county-level hospitals and creating remote ECG networks (16).

However, challenges have emerged during the implementation of these initiatives. Notably, the construction of medical consortiums has been criticized for being largely formalistic, with substantial deficiencies in grassroots healthcare capacity. Issues such as uneven resource distribution, a shortage of skilled personnel, and limited technological capabilities have hindered progress. Additionally, geographical constraints, including inconvenient transportation and significant distances from urban centers, have contributed to a reluctance among high-quality experts to relocate to these areas.

In 2019, Changshu City in Jiangsu Province was designated as one of the 567 “Tightly Integrated County-level Medical Community Pilot Counties” in China. Following the implementation of a new round of comprehensive healthcare reforms, the Changshu TCM Hospital, under the guidance of the Municipal Healthcare Reform Office and the Municipal Health Commission, signed regional and specialty medical community agreements with 20 primary healthcare institutions. Through initiatives such as expert team support, the development of demonstration outpatient services, the establishment of collaborative treatment groups in hospital wards, and the creation of pilot specialty alliances, the hospital has significantly promoted the construction of TCM specialty services at the grassroots level and enhanced the regional capacity and quality of TCM healthcare services, thereby fostering a mechanism for the collaborative development of regional TCM healthcare and wellness (17).

In summary, the integrated medical care vigorously developed by foreign typical countries and the HDT and medical community construction advocated in China have similarities. Firstly, they share a common objective of enhancing the efficiency of healthcare resource services, achieving rational allocation of medical resources, and guaranteeing the universality and sustainability of healthcare services for the population. Secondly, both models clearly and reasonably position hospitals at distinct levels, reflecting the HDT system. Thirdly, based on national conditions and socioeconomic development levels, tailored medical service models have been devised to better meet the diagnosis and treatment needs of diverse population segments.

### 2.3 Overview of TCM healthcare in Wuyi County

The county’s TCM hospital serves as the cornerstone for local TCM development. The hospital, covering approximately 10 acres, is equipped with 251 beds. By 2023, it had established one provincial-level key TCM specialty in rehabilitation and two county-level specialties in nephropathy and orthopedics. In 2019, the TCM hospital led the creation of a medical community by collaborating with seven township health centers. Prior to October 2022, only seven of the 19 township medical institutions within the county had dedicated TCM departments. In 2023, a shared Chinese medicine pharmacy was established countywide. As of the end of 2023, the county had a population of 469,000 and 2,852 beds, with a bed-to-population ratio of 6.08 beds per 1,000 individuals. Licensed (assistant) TCM practitioners numbered 224, which corresponds to a density of 0.48 practitioners per 1,000 people, with a significant concentration at the county level. TCM practitioners represented only 12.3% of the medical staff in the 19 grassroots healthcare institutions in the county. Furthermore, the implementation of TCM services that leverage the distinctive advantages of TCM remains limited. Existing service offerings are typically monotonous. The application of TCM’s appropriate technologies at the grassroots level is notably underdeveloped. According to data from the local Health and Family Planning Bureau, as of October 2022, the TCM service rate in grassroots healthcare institutions was merely 1%.

Despite the national government’s vigorous support for TCM and the growing demand from the public, there exists a marked contrast between the insufficient quantity of TCM resources in the county and their uneven distribution, compounded by underdevelopment. Addressing this imbalance and ensuring residents have access to high-quality, diverse TCM healthcare services in their local communities become a critical challenge.

## 3 Analysis of causes

### 3.1 Deficiency of TCM talent and underdevelopment of TCM disciplines

TCM holds considerable promise within hospitals at the city, county, and township levels. However, the weakening or even absence of TCM departments and integrated traditional Chinese and Western medicine departments is prevalent. TCM service capabilities across various hospital levels remain inferior. The integration of TCM with Western medicine has become commonplace, leading to the marginalization of traditional TCM concepts and methods. With the swift advancement of modern medicine and the prevalence of Western medical principles, some practitioners have diverged from the core values and technical frameworks of TCM, neglecting TCM’s holistic philosophy and the advantages inherent in syndrome differentiation and treatment. Consequently, the “Westernization” of TCM practitioners has emerged as a widespread and pronounced issue, leading to a shortage of professional TCM personnel and the weakening of TCM services within grassroots healthcare institutions. Field surveys reveal insufficient organizational management and financial investment in TCM departments. Additionally, there is a lack of emphasis on the development of TCM culture, and the distinctiveness of TCM remains underrepresented. These factors collectively hinder the development of TCM disciplines, medical care quality, TCM research, and educational capabilities.

### 3.2 Inadequate supervision of Chinese medicine pharmacies in remote mountainous areas

In remote mountainous regions, Chinese medicine pharmacies are widespread. However, the difficult transportation conditions impede effective regulatory oversight. Regulatory bodies at the grassroots level face limited human resources, financial constraints, and technological deficits, which hinder the deployment of specialized testing equipment or the training of dedicated regulatory personnel. This lack of infrastructure leads to insufficient supervision in critical areas such as the quality of Chinese medicinal materials and storage conditions. Many pharmacies in these areas rely on external procurement of medicinal materials without scientifically rigorous acceptance standards and traceability mechanisms, rendering them susceptible to the influx of substandard materials. Furthermore, the management structures of these pharmacies are often disorganized, with some falling under the jurisdiction of Western medicine pharmacies, TCM departments, or third-party contractors. This fragmentation results in “multiple supervisory bodies” or even “regulatory vacuums.”

### 3.3 Low social awareness

Despite the long history and profound cultural heritage of TCM in China, its awareness among the general public in modern society is limited. In more remote regions, the dissemination and popularization of Traditional Chinese Medicine (TCM) are increasingly inferior. A segment of the population remains skeptical toward TCM, questioning its therapeutic efficacy and safety. The complex preparation processes of Chinese herbal decoctions, their inherently bitter taste, and their relatively slow onset of effects often lead individuals to forgo TCM in favor of Western medical treatments. This trend undermines public acceptance and utilization of TCM, consequently retarding the transmission and advancement of TCM culture.

## 4 Strategies

### 4.1 Construction of shared Chinese medicine pharmacies

In order to tackle the issues of uneven TCM resource distribution and inadequate regulatory oversight of Chinese medicine pharmacies in remote mountainous areas, this study introduced a model inspired by the cloud-based shared Chinese medicine pharmacy system implemented in Xiangshan County. In October 2023, the county established a “Shared Chinese Medicine Pharmacy” under its medical consortium. By incorporating centralized procurement, standardized processing, and unified distribution, this initiative effectively reduces procurement costs, guarantees the quality of medicinal materials, and enables the efficient provision of services through a model of “online prescriptions and home delivery.”

### 4.2 Strengthening the construction of TCM talent pool

In 2024, a series of training initiatives were conducted under the leadership of county-level TCM hospitals, aiming to enhance the quality of the TCM workforce and reinforce both the training and recruitment of TCM professionals. Six specialized training courses were held, training over 500 healthcare professionals and cultivating 60 key personnel in appropriate TCM technologies and nursing. A 2024 training program on the promotion of appropriate TCM techniques for rural doctors was conducted, with 80 rural doctors participating in the training and assessment. Additionally, a training session for key personnel at grassroots appropriate TCM technique promotion sites was organized with 24 participants.

Regarding talent development, on the one hand, the local government introduced policies to support the professional development of in-service healthcare workers, offering financial incentives for those pursuing advanced degrees and enhancing the overall quality of TCM staff training. On the other hand, a full-time graduate training model was implemented, signing agreements with enrolled graduate students and offering stipends to cover tuition and living expenses during their studies. Upon completion of their studies, these graduates were recruited through campus recruitment efforts and employed in TCM-related roles, thus attracting high-caliber talent to the TCM field. In 2024, seven graduate students were recruited through this model. Moreover, a collaborative partnership with Jinhua Vocational and Technical College was established, providing educational opportunities for 19 students, who subsequently contributed to the development of grassroots TCM services upon graduation. Furthermore, initiatives were implemented to encourage practitioners of Western medicine to engage in the study of TCM, thereby strengthening the professional competence and skill sets of healthcare professionals in the field of TCM.

### 4.3 Construction of medical alliances and communities in the county

Drawing on the successful case of Ningxiang City in Hunan Province, the county has actively promoted the construction of medical consortia and community-based health organizations. The county-level TCM hospital serves as the leading entity, collaborating with township health centers and village clinics to form a medical community and establishing close cooperative relationships among these institutions (18). Through regular business guidance, technical training, two-way referrals, and other methods, the TCM service capabilities of grassroots medical institutions are enhanced, enabling the public to enjoy high-quality TCM services at their doorstep. The county-level TCM hospitals have established a medical consortium with Hangzhou TCM Hospital, where the higher-level hospital regularly sends experts to the grassroots institutions for outpatient consultations, research, and teaching activities. This collaboration seeks to enhance the professional skills and diagnostic capabilities of local healthcare staff.

### 4.4 Construction of specialized alliances

The county-level TCM hospitals, in collaboration with Hangzhou TCM Hospital, have built two specialized TCM alliances (the TCM Nephrology Alliance and the TCM Gynecology Alliance). These alliances aim to foster deeper internal communication and cooperation, boost research innovation, and strengthen talent development, ultimately improving the quality of TCM services available to the local population.

Given the uneven distribution of TCM resources within the county, the “Wuyi mode” integrates the “Medical Consortium + Community-based Health Organization + Specialized Alliances + Shared Chinese Medicine Pharmacies.” This model facilitates the development and application of medical talent and creates an integrated TCM service network that addresses the limitations of single-model approaches. For example, the combination of “Medical Consortium + Community-based Health Organization” involves collaboration with provincial and municipal tertiary hospitals to construct specialized TCM alliances. Experts are regularly deployed to guide grassroots staff, thereby enhancing the TCM service capabilities at the local level. Meanwhile, through the utilization of the “County-level Shared Chinese Medicine Pharmacy” network, the full range of TCM services—from diagnosis and prescription review to the preparation and delivery of herbal medicines—can be completed at the village level. This ensures that minor illnesses can be addressed without requiring individuals to leave their village, and herbal medicines are delivered directly to their homes. According to data from the local health authority’s official website, in 2024, the “Wuyi mode” facilitated 55,879 visits to shared Chinese medicine pharmacies, reflecting a 51.48% increase compared to 2023. The total number of TCM service visits reached 60,071, a 35.4% year-on-year growth.

Comprehensive application of innovative models such as county-level medical alliances, medical communities, specialized alliances, and shared Chinese medicine pharmacies can promote the diffusion of “technology and services” to the public, coordinate the bidirectional flow of “personnel and referrals,” and solidify the integration of “specialties and public health,” constructing an integrated TCM service system within the county. It can effectively address the disparate distribution of TCM resources within the county. These models optimize the allocation and utilization of TCM resources while improving the overall efficiency and quality of TCM services.

## 5 Limitation

### 5.1 Constraints on resources and funding

These models seek to enhance service efficiency through resource integration and sharing. However, economically underdeveloped regions often face constraints due to funding shortages. Establishing and maintaining medical alliances and communities necessitate stable financial support and substantial initial investments, particularly for acquiring high-quality medical equipment and advanced technologies. In areas with limited resources, such high-cost investments may pose burdensome challenges.

### 5.2 Lagging policies and regulations

As medical alliances rapidly develop, existing policies and regulations may fail to keep up. Ambiguous policies and incomplete regulations may result in legal hurdles in the practical operation of these models, impeding their efficiency and scalability.

### 5.3 Unevenness in technology and service quality

Although medical alliances aim to elevate the standard of medical services within the county by deploying technology and services, high-quality medical services and expert resources may be concentrated in specific alliance centers, resulting in unbalanced distribution of service quality and technological levels.

### 5.4 Personnel training and retention

In remote areas, training and retaining medical personnel is a long-standing issue. Despite the contribution of medical alliances and specialized alliances to technology transfer and knowledge dissemination, attracting and maintaining excellent medical talents, particularly experts in the field of TCM, remains a challenge.

### 5.5 Public awareness and acceptance

Efforts to strengthen publicity and foster public understanding of TCM notwithstanding, the traditional nature of TCM and individual variations in treatment outcomes may engender skepticism or reservations among certain segments of the public. Cultivating broader acceptance and trust requires sustained educational initiatives and time.

Comprehensive strategies spanning various domains within the county are imperative to surmount these limitations. These encompass continual refinement and innovation of existing models, bolstering policy backing and financial support, updating pertinent policies and regulations, intensifying personnel training initiatives and talent acquisition mechanisms, optimizing resource allocation, and promoting robust development of TCM within the county. Meanwhile, heightening publicity and advocacy to augment public awareness and confidence in TCM can foster its expanded role within the county.

## 6 Conclusion

Although innovative approaches such as medical consortia, medical communities, specialist alliances, and shared Chinese medicine pharmacies have specific limitations, they offer novel concepts and avenues for addressing the unequal distribution of TCM resources in county settings, forming localized medical plates. Emphasis can be placed on refining the TCM management framework within county-level medical communities, establishing a shared platform for TCM information exchange, constructing comprehensive talent pipelines for TCM in township health centers, heightening the dissemination of appropriate technologies in TCM, coordinating infrastructure investments in county-level medical communities, and conducting cultural outreach through diverse channels and mediums. By constantly optimizing and perfecting these models, the robust development of TCM can be propelled, thus contributing to the realization of a healthy China.
